# Understanding the connection: attachment patterns and interpersonal competence in nursing undergraduates

**DOI:** 10.1186/s12912-025-03425-x

**Published:** 2025-07-04

**Authors:** Mona Metwally El-Sayed, Samah Mohamed Taha, Eman Sameh AbdElhay, Bothaina Hussein Hassan, Helalia Shalabi Mohamed Shalabi, Mahmoud Abdelwahab Khedr

**Affiliations:** 1 Psychiatric Nursing and Mental Health, Faculty of Nursing, Alexandria University, Alexandria, Egypt; 2 Nursing and Mental Health, Faculty of Nursing, Mansoura University, Mansoura, Egypt; 3 Faculty of Nursing, Alexandria University, Alexandria, 21527 Egypt; 4 Community Health Nursing, Cairo University, Giza, Egypt

**Keywords:** Humans, Interpersonal relations, Students, Nursing

## Abstract

**Background:**

Interpersonal skills are vital in nursing and influence patient care and teamwork. Attachment patterns strongly affect these skills, with secure attachments fostering empathy and effective communication.

**Aim:**

This study examined the relationships between attachment patterns (secure, anxious, avoidant) and interpersonal competencies among nursing students.

**Methods:**

A cross-sectional self-report questionnaire, which included data from 426 undergraduate nursing students, was collected at Mansoura University in Egypt. Sociodemographic information, the Revised Adult Attachment Scale (RAAS), and the Social and Emotional Competencies Questionnaire (SEC-Q) were used for data collection.

**Results:**

Secure attachment was positively associated with self-awareness (*r* = 0.201, *p* ≤ 0.01), social awareness (*r* = 0.202, *p* ≤ 0.01), and the total SEC-Q score (*r* = 0.210, *p* ≤ 0.01). In contrast, dependent attachment exhibited a weak negative correlation with social awareness (*r* = -0.134, *p* ≤ 0.05), whereas anxious attachment was negatively correlated with decision-making (*r* = -0.144, *p* ≤ 0.05). Regression analysis revealed that attachment patterns significantly predicted social-emotional competency (F = 169.782, *p* < 0.001, R² = 0.168), accounting for 16.2% of the variance in the SEC-Q scores. Secure attachment positively predicted competency (B = 0.071, Beta = 0.191, t = 2.750, *p* = 0.006), whereas dependent attachment negatively predicted it (B = -0.100, Beta = -0.193, t = -2.775, *p* = 0.006). Anxious attachment had no significant effect (B = -0.209, Beta = -0.030, t = -0.419, *p* = 0.676). The Pearson correlation coefficient, multiple linear regression analysis, and mean and standard deviation were employed for statistical analysis.

**Conclusion:**

Nurturing secure attachment in nursing education can enhance students’ interpersonal competencies, ultimately improving patient care and team dynamics. Addressing attachment-related challenges helps foster emotional intelligence, benefiting both students and future patients.

**Clinical trial number:**

Not applicable.

## Introduction

Interpersonal competency is fundamental to effective nursing practice. It plays a crucial role in establishing therapeutic relationships with patients, collaborating with healthcare teams, and providing high-quality care. For nursing students, developing these competencies is essential, as they prepare to enter a profession that demands empathy, communication, and emotional intelligence [1, 2].

Research has consistently shown that individuals with secure attachments tend to have favourable views of themselves and others, allowing them to approach social interactions with confidence and openness [3, 4]. This improved their ability to connect with patients, understand their needs, and provide compassionate care [5].

Conversely, insecure attachment styles, such as anxious or avoidant patterns, may pose challenges in forming strong interpersonal relationship skills [5]. Nursing students who have insecure attachments may struggle with elements of patient care that demand emotional engagement or may find it difficult to build trusting relationships with colleagues and mentors [6, 7]. Recognizing these patterns can assist educators in customizing their strategies to help students navigate these potential barriers.

Attachment styles can affect how nursing students interact with their learning environment, peers, and clinical experiences within the educational setting [8]. Individuals with secure attachments are more likely to seek help when needed, engage in collaborative learning, and adapt more quickly to the challenges of clinical placements. This adaptability is essential in a field that demands continuous learning and flexibility [7, 9].

The impact of attachment styles extends beyond individual interactions to broader aspects of nursing practice. Nursing students with secure attachment may be better equipped to handle the emotional demands of the profession, demonstrating greater resilience in the face of stress and burnout. This emotional stability can lead to improved patient outcomes and increased job satisfaction in the long term [10].

## Theoretical background

Attachment theory, initially introduced by John Bowlby, proposes that early relationships with caregivers influence an individual’s capacity to develop and sustain relationships throughout their life [11, 12]. These early experiences shape internal working models that affect how individuals view themselves and others during social interactions [12]In nursing education, recognizing the influence of attachment styles on interpersonal skills can offer valuable insights into effectively preparing future healthcare professionals.

Attachment signifies an emotional bond between two people, marked by the expectation that one or both individuals will provide care and protection in times of need [13]. Bowlby characterized the attachment behaviors exhibited by infants as the primary mechanism used to elicit the proximity of a protective caregiver during periods of vulnerability [12]. Throughout their developmental trajectory, infants assimilate their formative experiences with caregivers, thereby constructing expectations regarding the caregiver’s benevolence and emotional availability in times of distress. These internalized cognitive representations of the self and others significantly influence their attachment styles in adulthood, affecting personal cognition, emotional regulation, interpersonal behavior, and responses to distressing experiences (e.g., separation, loss, perceived threat, rejection, illness) [5, 14]. Attachment theory offers a framework for educators, clinicians, and researchers to study how early experiences influence later life adjustments. It elucidates individual variations in emotional regulation, stress responses, and interpersonal behavior [13].

Recognizing the importance of attachment patterns, nursing education programs are increasingly integrating elements that promote secure attachment and improve interpersonal competencies [15]. Preceptorship models offer students the chance to build supportive relationships with experienced nurses, potentially reflecting secure attachment dynamics and fostering professional growth [16].

### Significance of the study

The COVID-19 pandemic has underscored the essential importance of robust interpersonal skills in healthcare while posing challenges to conventional skill development methods [1, 17]. Limitations in terms of clinical training time and fewer face-to-face interactions have necessitated innovative strategies for nurturing these essential competencies in nursing students [18, 19]. As the healthcare landscape evolves, establishing meaningful connections with patients and colleagues remains essential for effective nursing practices [20]. Attachment styles, which are deeply rooted in early experiences, play a significant role in shaping these abilities [21].

Research in this field is expanding, as recent studies have examined the mediating factors between attachment styles and empathy among nursing students [10, 22]. These investigations provide valuable insights into complex dynamics and offer potential avenues for enhancing interpersonal skill development in nursing education. The journey from a nursing student to a competent practitioner involves the acquisition of clinical knowledge and the cultivation of a deep understanding of human relationships [22]. Attachment theory provides a solid framework for understanding these interpersonal dynamics, shaping educational strategies and clinical practice [11].

As the demands of modern healthcare continue to evolve, the development of strong interpersonal competencies among nursing students has become increasingly critical. These competencies are closely linked to attachment styles, which influence the ability to build therapeutic relationships, communicate effectively, and function within multidisciplinary teams. While global research has underscored the importance of secure attachment and emotional intelligence in nursing education, there is a noticeable gap in the literature that specifically examines these dynamics within the context of Egypt and the broader MENA region. Few studies have explored how attachment patterns directly impact interpersonal competence among undergraduate nursing students in these settings. This study seeks to fill that gap by investigating the relationship between attachment styles and interpersonal skills among nursing students. The findings aim to inform targeted educational strategies that cultivate secure attachment and emotional intelligence, thereby enhancing students’ readiness for professional practice. Ultimately, improving these attributes may lead to better patient care, increased satisfaction, and more resilient, emotionally attuned healthcare providers.

### Research questions

1. What is the prevalence of different attachment patterns (secure, anxious, and dependent) among undergraduate nursing candidates?
2. Are there significant differences in interpersonal competence among nursing students with secure versus insecure attachment patterns?
3. Are there differences in the attachment patterns and interpersonal competence of nursing candidates based on specific demographic factors, such as gender and residence?

## Methods

### Study design and setting

This study utilized a cross-sectional survey from October to December 2024, adhering to the Strengthening the Reporting of Observational Studies in Epidemiology (STROBE) standard. It took place at the College of Nursing at Mansoura University, Egypt. The Nursing College comprises nine specialized scientific departments that span various nursing disciplines. This diverse departmental structure not only enhances academic experience but also enriches the educational offerings available to both national and international students. It provides undergraduate and graduate nursing programs using the credit hours system, which facilitates a structured approach for tracking academic progress and enables comprehensive evaluations of educational outcomes.

### Sample size and study participants

The study’s target population included undergraduate nursing students. To be eligible to participate, students were required to meet the following criteria: both genders, Egyptian nationality, current enrollment in the College of Nursing for the 2024–2025 academic year, and a willingness to participate. The participants were asked to provide a comprehensive medical history, which was reviewed for any identified mental health conditions. The exclusion criteria included any diagnosed mental health illnesses, such as depressive disorders, bipolar disorders, or anxiety-related disorders.

The sample size was established via the statistical methods provided in MedCalc Statistical Software version 18.2.1 (MedCalc Software BVBA, Ostend, Belgium; http://www.medcalc.org, viewed on October 1, 2024) [23]. To achieve adequate statistical power and accuracy, a Type I error rate (alpha) of 0.05 and a Type II error rate (beta) of 0.10 were used, corresponding to a statistical power of 90%, as indicated by previous studies from Asayesh et al. (2024), Salehi et al. (2020) and El-Sayed et al. (2024) [24–26]. On the basis of these parameters, the sample size necessary to detect a statistically significant difference was approximately 389 participants. To account for a potential nonresponse rate of 10% and attrition bias, the total targeted sample size was 437 participants, ensuring the study’s robustness and its ability to detect meaningful differences with high confidence.

### Recruitment and sampling method

**Fig. 1:**
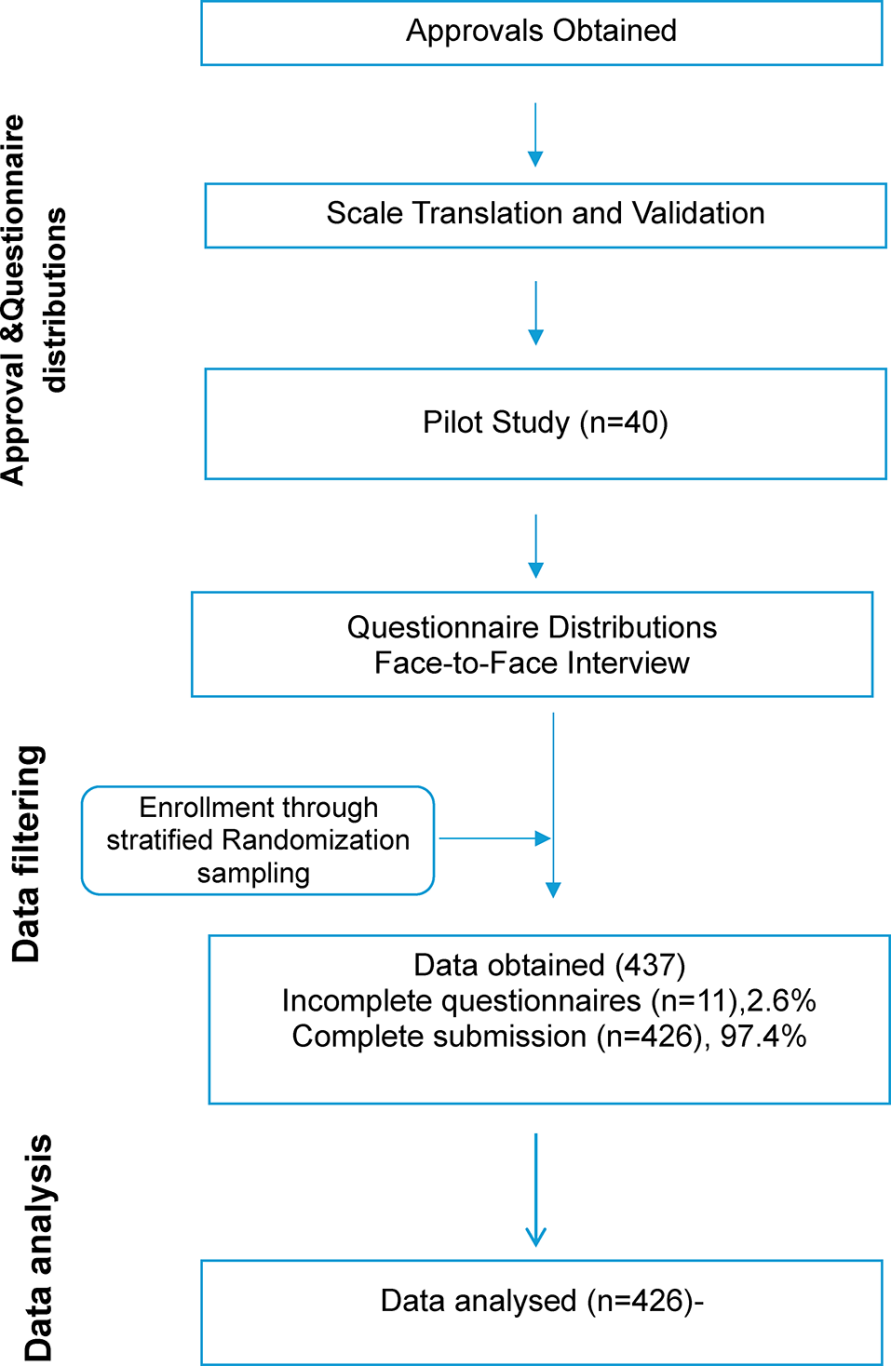
Flow chart of the data collection process

A stratified sampling technique was employed to ensure a representative sample from each academic year. Students in each academic year were also categorized by semester, and a proportionate allocation method was utilized to select participants from each group. The Faculty of Nursing’s Student Affairs Department reported that 5,910 undergraduate students were enrolled in the academic year, distributed as follows: 1,742 in the first year, 1,497 in the second year, 1,237 in the third year, and 1,434 in the fourth year. A total of 437 students from this group were invited to participate in the research. However, two students were deemed ineligible, five declined to participate, and three withdrew during the study, resulting in a response rate of 97.4% and a dropout rate of 2.6%. The final sample consisted of 426 students selected through a stratified randomized process and proportional allocation method to ensure representativeness. The cohort included 121 students from the first year, 105 from the second year, 98 from the third year, and 102 from the fourth year (Fig. 1).

### Measurements of interest

#### Demographic data and structured sheet

The sheet provides detailed information about the participants’ demographic characteristics, including age, gender, marital status, living arrangements, academic year, and frequency of visiting relatives.

### Revised adult attachment scale (RAAS)

The RAAS is a widely used self-report questionnaire designed to assess adult attachment patterns, emphasizing how individuals think, feel, and behave in close relationships [27]. The RAAS includes 18 items divided into three subscales: Secure, which measures comfort with intimacy and emotional closeness; Dependent, which assesses trust and reliability in others; and Anxious, which evaluates fears of rejection and abandonment. The scoring system employs a 5-point Likert scale ranging from “not at all characteristic of me” to “extremely characteristic of me.” Each subscale contains six items, and the total score for a subscale is calculated by summing the responses to its corresponding items. The mean score is then derived by dividing this sum by six, with higher scores indicating a stronger tendency toward that attachment dimension. The Arabic version of the RAAS was used in a study that reported a Cronbach’s alpha of 0.76. This indicates good internal consistency for the scale within the sample studied [28]. This Arabic version was employed in this study.

### Social and emotional competencies questionnaire (SEC-Q)

The SEC-Q is a comprehensive self-assessment tool designed to evaluate an individual’s social and emotional skills across several domains [29]. It assesses competencies such as self-awareness, self-regulation, social awareness, relationship skills, and responsible decision-making. The respondents evaluate themselves on a series of items via a Likert scale, typically ranging from 1 (strongly disagree) to 5 (strongly agree), allowing for a nuanced understanding of their ability to manage emotions, build relationships, and effectively navigate social interactions. The scoring system sums the scores for items related to each competency domain, yielding subscale scores that reflect strengths or areas for development within those domains. A higher total score in a specific domain indicates enhanced proficiency in that competency. The scale showed acceptable reliability across all the subscales (Cronbach’s α: self-awareness: 0.64, social awareness: 0.72, self-management: 0.73, relationship management: 0.69, responsible decision-making: 0.76, and overall scale: 0.86) [30]. The scale was translated into Arabic, and its validity was rigorously assessed via exploratory and confirmatory factor analyses. The Kaiser‒Meyer‒Olkin (KMO) measure demonstrated excellent sampling adequacy, with a value of 0.96. Exploratory factor analysis revealed that all the items accounted for 66.32% of the total variance, with factor loadings ranging from 0.76 to 0.89. Confirmatory factor analysis confirmed the structural integrity of the scale, yielding fit indices within acceptable ranges (χ²/df = 3.15, RMSEA = 0.007, CFI = 0.931, TLI = 0.925). Additionally, the scale exhibited high reliability, as indicated by a Cronbach’s alpha coefficient of 0.87.

### Procedure

#### Ethical approval

The study proposal was reviewed and approved by the Research Ethics Committee (REC) of the Faculty of Nursing at Mansoura University, Egypt, under ethical clearance reference number (**IRB**/000523). All research procedures were conducted in strict accordance with the ethical principles outlined in the Declaration of Helsinki and its subsequent revisions, ensuring compliance with internationally accepted standards for research involving human participants. Before participation, all individuals received detailed verbal and written information regarding the objectives, methodology, potential risks and benefits, as well as the voluntary nature of their involvement. Informed consent was obtained from each participant after verifying their clear understanding of the study’s content and their rights as research subjects. Emphasis was placed on voluntary participation, and participants were assured that their involvement was entirely optional, with the right to withdraw from the study at any time without providing a reason and without any repercussions. Confidentiality and anonymity were rigorously maintained throughout the data collection, analysis, and reporting. Personal identifiers were removed or anonymized to protect participants’ privacy, and all collected data were securely stored in accordance with institutional and ethical data protection protocols.

#### Preliminary study

A preliminary study was conducted involving 40 participants who were randomly selected from the academic affairs database via the Research Randomizer tool (version 0.4). The primary objective was to evaluate the clarity, relevance, and applicability of the study instruments. The results demonstrated that all the questions were clear, comprehensible, and free of ambiguity, as no participants reported any confusion. Moreover, the questions were suitable for effectively measuring participants’ attachment patterns and interpersonal competencies. The participants also noted that the length and order of the questionnaires were both appropriate and easy to follow. Additionally, the random selection process was executed without any technical issues. On the basis of these findings, the study instruments were well designed and required no modifications. This preliminary assessment confirmed the validity, reliability, and practicality of the scales, ensuring their appropriateness for the main study.

#### Data collection

The study began by obtaining the necessary approvals and collecting Excel spreadsheets from the academic affairs department, which listed all the undergraduate students. The participants were selected via a random generator set to 0.4 to ensure a fair sample from each school year. This selection process was repeated until the desired number of students was obtained. Before the data were collected, the researchers explained the study’s objectives to each participant, emphasizing that participation was voluntary. Informed written consent was obtained from all participants as a prerequisite for their involvement. The participants were assured that their responses would remain confidential to build trust. The surveys were administered in quiet, distraction-free settings, such as empty lecture halls and libraries, between 9:00 AM and 2:00 PM from Sunday to Thursday. Each participant required approximately 15 to 20 min to complete the questionnaire.

#### Data analysis

The gathered data were examined via the SPSS program, version 26. Descriptive statistics, including frequencies and percentages, were employed to describe categorical variables, providing an overview of response distributions among categories. The mean (M) and standard deviation (SD) were calculated for continuous variables to characterize the central tendency and variability of the data. The Pearson correlation coefficient (r) assesses the strength and direction of correlations among continuous variables. Multiple linear regression analysis was conducted to evaluate the impact of attachment patterns on interpersonal competencies. Statistical significance was determined using an alpha (α) level of 0.05, with p values below this threshold considered statistically significant. This indicated a minimal likelihood that the observed outcomes resulted from chance.

## Results

Table 1 Shows that most participants were female (62.9%) and aged between 18 and 20 years (35.4%). In terms of marital status, 79.4% were single, and 20.6% were married, with no participants indicating that they were divorced or widowed. A significant proportion lived in urban areas (76.1%), whereas 23.9% resided in rural areas. With respect to living arrangements, 73.2% lived with family members, 15.5% with relatives or friends, and 11.3% on university campuses. The participants were spread across years of study, with 29.6% in their first year, 25.1% in their second year, 21.6% in their third year, and 23.7% in their fourth year. Regular visits to relatives were reported by 59.1% of the participants, whereas 40.9% did not visit relatives regularly. Furthermore, 62.9% of the participants worked while studying, whereas 37.1% did not.

**Table 1:** Distribution of the demographic characteristics of the participants (*n* = 426)

Demographic Characteristics	*N* = 426	%
Gender		
Male	158	37.1
Female	268	62.9
Age		
18 < 20	151	35.4
20 < 22	117	27.4
22 < 24	89	20.8
≥ 24	69	16.4
Marital Statues		
Single	338	79.4
Married	88	20.6
Divorced or widowed	0	0.0
Region of Residence		
Urban	324	76.1
Rural	102	23.9
Living Arrangements		
Family Members	312	73.2
Relatives or Friends	66	15.5
University Campus	48	11.3
Year of studying		
First	126	29.6
Second	107	25.1
Third	92	21.6
Fourth	101	23.7
Regular Visiting the Relatives		
Yes	252	59.1
No	174	40.9
Working while studying		
Yes	268	62.9
No	158	37.1

Table 2 displays the mean scores for the RAAS. The results revealed that participants scored highest in the secure attachment dimension (M = 3.22, SD = 0.60), followed by the anxious attachment dimension (M = 2.91, SD = 0.85) and the dependent attachment dimension (M = 2.78, SD = 0.58), resulting in a total RAAS score of 8.91 (SD = 2.03). For the SEC-Q, the participants presented the highest mean scores for social awareness and prosocial behavior (M = 24.30, SD = 4.46), followed by self-awareness (M = 15.39, SD = 3.33), self-management and motivation (M = 11.62, SD = 2.74), and decision-making (M = 11.34, SD = 2.96), resulting in a total SEC-Q score of 51.76 (SD = 9.88).

**Table 2:** The mean scores of the SEC-Q and RAAS among the participants (*n* = 426)

	Variables	M (SD)	
RAAS	Secure	3.22 (0.60)	
	Dependent	2.78 (0.58)	
	Anxious	2.91 (0.85)	
	Total of RAAS	8.91 (2.03)	
SEC-Q	Self-awareness	15.39 (3.33)	
	Self-management and motivation	11.62 (2.74)	
	Social awareness and prosocial behavior	24.30 (4.46)	
	Decision-making	11.34 (2.96)	
	Total of SEC-Q	51.76 (9.88)	

SEC-Q: Social and Emotional Competencies Questionnaire

RAAS: Revised Adult Attachment Scale

M: Mean; SD: Standard deviation

Table 3 shows that secure attachment was positively associated with self-awareness (*r* = 0.201, *p* ≤ 0.01), social awareness, prosocial behavior (*r* = 0.202, *p* ≤ 0.01), and the total SEC-Q score (*r* = 0.210, *p* ≤ 0.01), highlighting the connection between secure attachment and emotional and social competencies. In contrast, the dependent attachment style exhibited weak negative correlations with social awareness and prosocial behavior (*r* = -0.134, *p* ≤ 0.05), whereas the anxious attachment style had a negative correlation with decision-making (*r* = -0.144, *p* ≤ 0.05). The total RAAS score was positively associated with all SEC-Q dimensions, especially social awareness and prosocial behavior (*r* = 0.322, *p* ≤ 0.01), indicating that stronger attachment patterns, particularly secure attachment, are linked to greater emotional competence.

**Table 3:** Correlation coefficients between the SEC-Q score and RAAS score among the participants (*n* = 426)

SEC-Q		SEC-Q					RAAS			
		Self-awareness	Self-Management and Motivation	Social awareness and prosocial behavior	Decision-making	Total of SEC-Q	Secure	Dependent	Anxious	Total of RAAS
Self-awareness	r									
	p									
Self-management and Motivation	r	0.261^**^								
	p	0.001								
Social awareness and prosocial behavior	r	0.364^**^	0.251^**^							
	p	0.001	0.001							
Decision-making	r	0.341^**^	0.230^**^	0.541^**^						
	p	0.001	0.000	0.001						
Total of SEC-Q	r	0.426^**^	0.281^**^	0.478^**^	0.621^**^					
	p	0.000	0.001	0.000	0.000					
Secure	r	0.201^**^	0.140^*^	0.202^**^	0.126	0.210^**^				
	p	0.000	0.040	0.000	0.201	0.001				
Dependent	r	-0.093	-0.054	-0.134^*^	-0.080	-0.117	0.462^**^			
	p	0.265	0.387	0.020	0.311	0.243	0.001			
Anxious	r	0.006	-0.020	0.012	-0.144^*^	-0.036	0.521^**^	0.416^**^		
	p	0.268	0.424	0.121	0.031	0.310	0.001	0.001		
Total of RAAS	r	0.236^**^	0.214^**^	0.322^**^	0.142^**^	0.248	0.368^**^	0.369^**^	0.671^**^	
	p	0.001	0.002	0.001	0.001	0.001^**^	0.000	0.000	0.000	

r: Pearson correlation coefficient; *Statistically significant at *p* ≤ 0.05 (2-tailed); **Statistically significant at *p* ≤ 0.001 (2-tailed)

SEC-Q: Social and Emotional Competencies Questionnaire; RAAS: Revised Adult Attachment Scale

Table 4 shows that married participants scored significantly higher in the dependent attachment style (M = 3.12, SD = 0.67) than single participants did (M = 2.76, SD = 0.57, t = 2.088, *p* ≤ 0.05). Compared with males, females had slightly higher scores for secure attachment (M = 3.24, SD = 0.64) and social-emotional competency (M = 52.46, SD = 9.58). Compared with urban residents, rural participants scored higher in anxious attachment (M = 3.16, SD = 0.99) and social-emotional competency (M = 52.86, SD = 9.02); however, these differences were not statistically significant. The participants living with relatives achieved the highest secure attachment (M = 3.48, SD = 0.60) and social-emotional competency (M = 54.50, SD = 7.17), whereas those on campus had the lowest competency scores (M = 49.09, SD = 12.36). Regular family visits and working while studying were linked to slightly higher scores for secure attachment and social-emotional competency, but these differences also lacked statistical significance.

**Table 4:** Relationships between demographic characteristics, SEC-Q scores, and RAAS scores among participants (*n* = 426):

Variable		RAAS						SEC-Q	
		**Secure**		**Dependent**		**Anxious**		**M**	**SD**
		**M**	**SD**	**M**	**SD**	**M**	**SD**		
Gender	Male	3.18	0.48	2.82	0.54	2.89	0.83	49.72	10.53
	Female	3.24	0.64	2.77	0.60	2.92	0.86	52.46	9.58
t test		0.718		0.623		0.266		1.826	
Marital status	Single	3.22	0.60	2.76	0.57	2.91	0.84	51.57	9.75
	Married	3.26	0.62	3.12	0.67	3.04	1.13	55.08	11.93
t test		0.236		2.088*		0.537		1.200	
Region of Residence	Urban	3.21	0.60	2.79	0.59	2.89	0.84	51.64	9.98
	Rural	3.31	0.64	2.74	0.52	3.16	0.99	52.86	9.02
t test		0.156		0.195		1.473		0.010	
Living Arrangement	Family member	3.20	0.60	2.79	0.60	2.92	0.86	51.89	9.82
	University Campus	3.58	0.68	2.91	0.36	2.64	0.59	49.09	12.36
	Relatives	3.48	0.60	2.43	0.40	2.97	1.05	54.50	7.17
F test		2.192		1.463		0.417		1.267	
Work while studying	Yes	3.24	0.60	2.80	0.57	2.90	0.90	51.93	12.20
	No	3.22	0.61	2.78	0.59	2.92	0.84	51.69	8.92
t test		0.167		0.221		0.157		0.164	
Visiting the family members	Yes	3.26	0.56	2.81	0.56	2.92	0.89	52.41	8.79
	No	3.15	0.68	2.72	0.67	2.95	0.82	50.96	11.23
F test		0.700		0.507		0.465		1.151	

t test: independent samples t test; F test: ANOVA; *: Statistically significant at *p* ≤ 0.05 (2-tailed) **Statistically significant at *p* ≤ 0.001 (2-tailed); SEC-Q: Social and Emotional Competencies Questionnaire; RAAS: Revised Adult Attachment Scale

Table 5 presents the results of the multiple linear regression analysis that explored the impact of attachment patterns on social-emotional competency, revealing a statistically significant model (F = 169.782, *p* < 0.001) with an R² of 0.168. This finding indicates that attachment patterns accounted for 16.2% of the variance in the SEC-Q scores. Secure attachment positively predicted social-emotional competency (B = 0.071, Beta = 0.191, t = 2.750, *p* = 0.006), whereas dependent attachment negatively predicted it (B = -0.100, Beta = -0.193, t = -2.775, *p* = 0.006). Anxious attachment did not have a significant effect on social-emotional competency (B = -0.209, Beta = -0.030, t = -0.419, *p* = 0.676). These findings indicate that secure attachment is associated with higher levels of social-emotional competency, while dependent attachment is related to lower levels, and anxious attachment makes no meaningful contribution.

**Table 5:** Multiple linear regression analysis of the effects of attachment patterns and interpersonal competence (*n* = 426)

	^b^ SEC-Q			t	*p*	95% CI	
	Unstandardized Coefficients		Standardized Coefficients				
	***B***	***S.E***	***Beta***				
						***LL***	***UL***
Constant	51.753	5.920	----	8.743	0.000^**^	0.210	0.720
^a^ Secure	0.071	1.138	0.191	2.750	0.006^**^	0.050	0.094
^a^ Dependent	-0.100	1.177	-0.193	-2.775	0.006^**^	-0.080	-0.169
^a^ Anxious	-0.209	0.822	-0.030	-0.419	0.676	-0.200	-0.261
	**R**^**2**^ **= 0.168**,** Adjusted R**^**2**^ **= 0.162**, F = 169.782^*^, *p* < 0.001^*^						

^a^ RAAS: Revised Adult Attachment Scale (independent variable)

^b^ SEC-Q: Social and Emotional Competencies Questionnaire (Dependent variable)

F, p: f and p values for the model R^2^: Coefficient of determination

B: Unstandardized Coefficients Beta: Standardized Coefficients

t: t test of significance LL: Lower limit UL: Upper limit

*: Statistically significant at *p* ≤ 0.05 (2-tailed) **Statistically significant at *p* ≤ 0.001 (2-tailed)

## Discussion

Interpersonal competencies are essential for nursing students, as they improve communication, teamwork, and patient care. These skills build trust with patients, foster effective collaboration with colleagues, and prepare students to handle the interpersonal demands of healthcare, ensuring compassionate and high-quality care. Exploring the impact of attachment patterns on interpersonal competencies provides a deeper understanding of how early relationships shape professional connections. It reveals how personal experiences influence nursing students’ interactions with patients and colleagues, ultimately enhancing their capacity to deliver compassionate, effective care in a healthcare environment [10]. Therefore, this study investigated the relationship between attachment styles and interpersonal competencies among nursing students.

Our results indicate that nursing students displayed the highest levels of secure attachment, followed by anxious and dependent attachment styles. These findings align with the findings of Moghadam et al., [31], who reported that secure attachment was the most prevalent attachment among medical students, whereas ambivalent attachment was the least common. They concluded that secure attachment helps medical students manage stress and that the higher rate of avoidant attachment in single individuals than in married individuals results from negative attitudes and challenges in forming relationships. Moreover, these findings contrast with those of Bordoagni et al.,, who reported that nursing students presented lower secure attachment and greater relationship anxiety than did the control group, with attachment anxiety being a significant predictor of burnout in nurses [32]. The study revealed that nursing students may compensate for attachment insecurity by improving their mentalization ability. It also proposed that attachment security could protect against burnout in professional nurses. Educational programs that enhance mentalization skills could facilitate the transition of nursing students into clinical practice, whereas strategies based on attachment theory might reduce burnout risk among nursing professionals.

Furthermore, Kaya examined the attachment styles of university nursing students and the factors influencing these styles [13]. She conducted a longitudinal follow-up with the Turkish School of Nursing regarding first-year students to evaluate changes in attachment styles. She reported a statistically significant decrease in insecure attachment styles by the end of the nursing program. Assessing attachment styles can be valuable for understanding and supporting nursing students. This aligns with our objective of exploring how attachment patterns influence interpersonal competencies among nursing students. By identifying changes in attachment styles throughout their education, we can gain better insight into how to support students during their training.

With respect to emotional and social competencies, our findings revealed that nursing students demonstrated the highest performance in social awareness and prosocial behavior, followed by self-awareness, self-management, motivation, and decision-making. This directly addresses our research question about the connection between attachment patterns and interpersonal competencies. Remarkably, students with secure attachment displayed greater social competence, indicating that fostering such attachment could be critical for effective patient care. Similarly, medical students show a stronger connection between prosocial behavior and career motivation than their peers in other fields do [26, 33]. The study proposed a model to encourage prosocial behavior on the basis of theories of planned behavior, self-determination, and social support. These findings underscore the essential role of family and school education in nurturing these behaviors throughout medical training, viewing them as crucial for developing professionalism and ensuring quality care in healthcare settings.

In this context, Eun Bin et al., emphasized that nurses engage in diverse interpersonal relationships that require constant interaction to maintain therapeutic connections [34]. This can lead to interpersonal conflicts, emotional strain, burnout, and increased turnover intentions. Therefore, nurses must have strong social-emotional competency, which involves effectively understanding and managing their emotions and those of others. This competency is crucial for building positive relationships, making responsible decisions, and thriving in the dynamic healthcare environment. Academic training focused on emotional development is essential in strengthening and preparing individuals for their professional lives. It enables them to navigate situations and conflicts effectively, fostering the competence expected of professionals. As a result, developing a critical perspective, intellectual curiosity, and communication skills is vital, particularly in socioprofessional interactions in nursing. This approach aims to improve nurses’ interpersonal relationships and emotional competencies, ensuring that they can provide compassionate, knowledgeable, and well-rounded care [35].

This study utilized the SEC-Q to assess social and emotional competencies across five domains: self-awareness, self-regulation, social awareness, relationship skills, and responsible decision-making. These results directly address our research questions regarding the prevalence of attachment patterns and their connection to interpersonal competencies. Notably, individuals with secure attachment often demonstrate a strong sense of self-worth, which enhances their ability to empathize and cooperate. Our findings indicated that secure attachment positively correlates with self-awareness (*r* = 0.201, *p* ≤ 0.01), social awareness, and prosocial behavior (*r* = 0.202, *p* ≤ 0.01), aligning with emotional and social competencies. In contrast, dependent attachment was weakly negatively correlated with social awareness and prosocial behavior (*r* = -0.134, *p* ≤ 0.05), whereas anxious attachment was negatively associated with decision-making (*r* = -0.144, *p* ≤ 0.05).

Furthermore, regression analysis confirmed that attachment patterns significantly predicted social-emotional competency, accounting for 16.2% of the variance in the SEC-Q scores. This underscores the importance of secure attachment in fostering not only emotional stability but also effective interpersonal skills, which are crucial in nursing practice. These findings highlight the role of secure attachment in the development of emotional and social skills and highlight the vulnerabilities associated with less secure attachment styles. This can be explained by the fact that individuals with secure attachment cultivate a strong sense of self-worth and emotional regulation, enabling them to better understand their own emotions and those of others. This emotional stability promotes empathy, cooperation, and prosocial behaviors, as they feel more confident in forming and maintaining healthy relationships.

In contrast, insecure attachment can hinder emotional awareness and restrict the ability to engage in prosocial behaviors. Moghadam et al. [31] explained that secure attachment activates Bowlby’s “discovery system,” allowing individuals to explore their environment and feel in control [12]. Over time, it fosters mastery, frustration management, emotional reflection, and positive beliefs about personal effectiveness. Traits associated with secure attachment, such as positive perfectionism, self-esteem, emotional regulation, and diminished stress, may act as protective factors for medical students, who frequently encounter high levels of stress. According to Mikulincer and Shaver, secure attachment reduces self-protection needs, allowing individuals to focus on others’ needs and engage in helpful actions [36].

Additionally, the care received from attachment figures acts as a model for supporting others. Secured individuals tend to have a positive self-concept, allowing them to manage their emotions effectively. In contrast, anxiously attached individuals concentrate on their distress, which limits their ability to attend to others. Avoidant individuals are less likely to offer help, as they feel uncomfortable with emotional closeness and hold unfavourable views of others [37]. These findings align with those of the study by Shi et al., which demonstrated that secure attachment is associated with prosocial behavior [38]. Furthermore, moral disengagement acts as a mediator in the connection between secure attachment and prosocial behavior. Moral identity also influences this mediating effect, demonstrating a more significant impact in individuals with a high moral identity than in those with a low moral identity. Similarly, Anwer et al. securely attach individuals who display positive traits such as self-awareness, confidence, independence, emotional intelligence, and strong interpersonal skills [39]. They effectively regulate emotions in social situations. In contrast, individuals with insecure attachment styles—fearful, dismissing, or preoccupied—tend to be defensive and emotionally inflexible, which can lead to strained interactions. Those with dismissal attachment may possess high self-worth but often devalue others, struggle with emotional regulation, and resist adapting to social demands, ultimately harming their relationships. McDaniel, This study examined the effects of attachment insecurity on the mental and physical health of 184 nursing students [40]. The results indicated that greater attachment insecurity is associated with elevated stress, diminished well-being, poorer overall health, and increased depressive symptoms. Further analysis revealed that attachment avoidance and stress are significant predictors of both depression and reduced well-being.

The current study revealed that married students scored significantly higher in dependent attachment than single students did. Compared with male participants, female participants presented slightly higher scores for secure attachment and social-emotional competency, whereas rural participants scored higher scores for anxious attachment and social-emotional competency than did urban residents; however, these differences were not statistically significant. These observations connect back to our research questions regarding demographic influences on attachment patterns. Understanding these nuances can guide targeted interventions in nursing education. The participants living with relatives demonstrated the highest levels of secure attachment and social-emotional competency, whereas those residing on campuses presented the lowest levels of competency. Regular family visits and working during studies were linked to marginally greater secure attachment and social-emotional competency, although these differences also lacked statistical significance. Kaya’s results support these findings [13].

These findings contrast with those of Petrowski et al.,, who reported that attachment style varies with age and relationship status [41]. The study revealed that older single individuals often displayed anxious attachments, whereas younger individuals in relationships presented greater attachment anxiety. Education also played a role, as more educated individuals experienced fewer dependency issues regardless of their relationship status, and single individuals were generally more educated. Furthermore, they discovered that attachment style accounted for only 1% of the variance in relationship status, whereas sociodemographic variables explained 40%, highlighting the greater influence of demographic factors.

### Strengths and limitations

The study provides insightful results. However, its cross-sectional design limits the ability to establish causal relationships between attachment patterns and interpersonal competencies. Self-reported measures may also introduce social desirability bias, potentially affecting the accuracy of responses. Furthermore, cultural factors influencing attachment styles and emotional competencies were not explicitly considered, which may limit their applicability across diverse populations. To address these limitations, future research could adopt longitudinal designs to track changes in attachment patterns and interpersonal competencies, reducing concerns about causality. The incorporation of qualitative methods such as interviews or focus groups could provide deeper insights into students’ experiences and perceptions. Additionally, integrating cultural considerations into future studies and interventions could ensure that attachment-based strategies in nursing education are tailored to diverse student backgrounds, enhancing their effectiveness.

## Conclusion and recommendations

This study highlights the critical influence of attachment patterns on the development of interpersonal competence among nursing undergraduates. These findings indicate that secure attachment is significantly linked to stronger emotional and social skills, establishing it as a foundational aspect for effective interpersonal functioning in nursing practice. Conversely, dependent attachment may impede the development of these essential competencies, emphasizing the potential risks associated with insecure relational patterns. Additionally, some demographic variables, such as marital status, gender, and living arrangements, were significantly associated with attachment and emotional skills. Nonetheless, these trends suggest that personal and contextual factors may subtly shape students’ relational development. Since fostering secure attachment within nursing education is not only beneficial but also essential, experimental and intervention-based studies are encouraged to evaluate the effectiveness of attachment educational strategies, including mentorship models, reflective practice, and emotion-focused communication training. Furthermore, integrating mixed-method approaches could offer richer insights into the subjective experiences of nursing students with different attachment styles. Qualitative data would enrich quantitative findings and deepen our understanding of the psychological and contextual factors that influence interpersonal development in nursing education.

### Relevance to nursing practices

Understanding the role of attachment patterns in interpersonal competencies is crucial for nursing practice, as effective communication, empathy, and teamwork are essential for patient care and professional collaboration. This study highlights that secure attachment is positively linked to self-awareness, social awareness, and overall social-emotional competency, which are fundamental for establishing therapeutic relationships with patients and fostering teamwork in clinical settings. Conversely, dependent and anxious attachment styles may hinder decision-making and social interactions, potentially affecting patient outcomes and workplace dynamics. By integrating attachment theory into nursing education, institutions can implement targeted training programs that promote self-awareness, emotional regulation, and interpersonal communication skills. Strategies such as mentorship programs, reflective practices, and emotional intelligence workshops can help nursing students develop secure attachment behaviors, enabling them to handle high-pressure situations confidently and empathetically. Additionally, emphasizing the importance of acknowledging and valuing diverse cultural backgrounds can strengthen trust and foster positive relationships with both patients and colleagues. Moreover, recognizing attachment-related challenges can inform personalized support interventions for students struggling with emotional and social competencies, ultimately enhancing their readiness for patient-centered care. By fostering secure attachment and social-emotional skills, nursing programs can better prepare students for the complexities of healthcare environments, improving patient satisfaction and interprofessional collaboration.

## Data Availability

Data will be available upon reasonable request from the corresponding author.
